# Olopatadine Suppresses the Migration of THP-1 Monocytes Induced by S100A12 Protein

**DOI:** 10.1155/MI/2006/42726

**Published:** 2006-02-06

**Authors:** Kazuya Kishimoto, Satoshi Kaneko, Kenji Ohmori, Tadafumi Tamura, Kazuhide Hasegawa

**Affiliations:** Pharmaceutical Research Center, Kyowa Hakko Kogyo Co, Ltd, 1188 Shimotogari, Nagaizumi-cho, Sunto-gun, Shizuoka 411-8731, Japan

## Abstract

Olopatadine hydrochloride (olopatadine) is an antiallergic drug with histamine H_1_ receptor antagonistic activity. Recently, olopatadine has been shown to bind to S100A12 which is a member of the S100 family of calcium-binding proteins, and exerts multiple proinflammatory activities including chemotaxis for monocytes and neutrophils. In this study, we examined the possibility that the interaction of olopatadine with S100A12 inhibits the proinflammatory effects of S100A12. Pretreatment of olopatadine with S100A12 reduced migration of THP-1, a monocyte cell line, induced by S100A12 alone, but did not affect recombinant human regulated upon activation, normal T cell expressed and secreted (RANTES)-induced migration. Amlexanox, which also binds to S100A12, inhibited the THP-1 migration induced by S100A12. However, ketotifen, another histamine H_1_ receptor antagonist, had little effect on the activity of S100A12. These results suggest that olopatadine has a new mechanism of action, that is, suppression of the function of S100A12, in addition to histamine H_1_ receptor antagonistic activity.

## INTRODUCTION

S100A12 belongs to the S100 family of EF-hand-type calcium-binding proteins [1]. These proteins are involved in the regulation of a variety of intracellular activities, including protein phosphorylation, enzymatic activities, Ca^2+^ homeostasis, and intermediate filament polymerization [2]. On the other hand, several S100 proteins including S100A12 are secreted and have extracellular activities [2]. S100A12 belongs to a subset of the S100 protein family, which is termed the myeloid-related proteins (MRPs) [3–5], because expression of these proteins is almost completely restricted to cells of myeloid origin, such as neutrophils and monocytes. MRPs have proinflammatory effects in the extracellular milieu [6, 7]. S100A12 is detected in the synovial fluid and plasma of patients with gout, rheumatoid arthritis, and psoriatic arthritis [8], suggesting that S100A12 may be involved in the inflammation in these diseases.

S100A12 from bovine lung extract was identified as a ligand for the receptor for advanced glycation end products (RAGE), which is expressed on macrophages, endothelium, and lymphocytes. Endothelial cells incubated with S100A12 exhibited increased expression of intercellular adhesion molecule-1 and vascular cell adhesion molecule-1 through the activation of nuclear factor-κ B [9]. In addition, S100A12 induced the expression of tumor necrosis factor-α and interleukin-1β in a murine macrophage cell line [8]. Footpad injection of bovine S100A12 into mice resulted in the infiltration of leukocytes, and anti-S100A12 antibody blocked leukocyte recruitment in murine models of delayed-type hypersensitivity induced by methylated bovine serum albumin [9]. Furthermore, human recombinant S100A12 has been reported to be chemotactic for neutrophils and monocytes in vitro and in vivo [10, 11]. Thus, S100A12 and RAGE are attractive targets for the treatment of inflammation.

It has been reported that anti-allergic drugs/mast cell stabilizers such as amlexanox and cromolyn specifically bind to S100A12 and S100A13 which are another member of the S100 protein family [12]. It has also been reported that amlexanox repressed the fibroblast growth factor 1 (FGF-1) release induced by S100A13 [13, 14]. Therefore, it is expected that binding of these drugs to S100A12 result in the inhibition of its function, which may contribute to antiallergic or anti-inflammatory effects of these drugs.

Olopatadine hydrochloride (olopatadine: (Z)-11-(3-Dimethylaminopropylidene)-6,11-dihydrodibenz [b,e]oxepin-2-acetic acid monohydrochloride, ALLELOCK^®^, Kyowa Hakko Kogyo Co, Ltd, Japan) is an antiallergic agent with histamine H_1_ receptor antagonistic action that is indicated for the signs and symptoms of allergic rhinitis, chronic urticaria, eczema dermatitis, prurigo, pruritis cutaneous, psoriasis vulgaris, and erythema exsudativum multiform [15]. Olopatadine exhibits potent antihistamine activity in vivo following its systemic administration. In addition to its potent antihistaminic effect, previous studies have demonstrated that olopatadine inhibits the release of inflammatory lipid mediators such as leukotrienes and thromboxanes from human polymorphonuclear leukocytes and eosinophils [15]. Olopatadine also reduces the tachykinin release from peripheral sensory nerve endings [16]. Olopatadine inhibits eosinophil infiltration in both rat allergic rhinitis and mice chronic contact hypersensitivity models [17, 18]. However, the precise mechanism of inhibitory effects of olopatadine on infiltration of leukocytes is still unclear.

Recently, Okada et al have found that olopatadine binds specifically to S100A12 and S100A13 in a calcium-dependent manner as amlexanox and cromolyn do [19]. In the present study, we examined the inhibitory effect of olopatadine on the function of recombinant human S100A12.

## MATERIALS AND METHODS

### Materials

Olopatadine hydrochloride (olopatadine) was synthesized in Yokkaichi Plant, Kyowa Yuka Co, Ltd (Mie, Japan). Ketotifen fumarate (ketotifen) was purchased from Sigma Chemical (St Louis, Mo, USA). Amlexanox was extracted from SOLFA^®^ tablets purchased from Takeda Chemical Industries Inc (Osaka, Japan). These drugs were dissolved in DMSO. Recombinant human regulated upon activation, normal T cell expressed and secreted (RANTES) was purchased from R&D SYSTEMS (Minneapolis, Minn, USA).

### Recombinant S100A12 protein

Human S100A12 cDNA was a gift from Dr Ryoji Kobayashi (Department of Signal Transduction Sciences, Kagawa University Faculty of Medicine, Kagawa, Japan). Recombinant protein was expressed in E coli strain BL21 (DE3) and purified as previously described by Yamashita et al [20], with some modifications. Briefly, 300 mL DE3 culture was grown at 37°C to an OD600 of 0.6–0.8 and induced with 1 mmol/L isopropyl-β-D-thiogalactopyranoside for 4 h at 37°C. The cell pellet was resuspended in hypotonic buffer, 10 mmol/L Tris-HCl (pH 7.5) containing 1 mmol/L ethylenediaminetetraacetic acid, and then centrifuged at 10 000 g for 60 min. The supernatant was dialyzed against 20 mmol/L Tris-HCl (pH 7.5) (buffer A), and loaded onto a HiPrep 16/10 Q XL column (Amersham Biosciences, Piscataway, NJ, USA) equilibrated with buffer A. The protein was eluted with a linear gradient from 0 to 250 mmol/L NaCl. Fractions containing the protein were pooled and loaded onto a HiLoad 16/60 Superdex 75 pg (Amersham Biosciences) equilibrated with buffer A. Fractions containing the protein were pooled and further purified using a Mono Q HR 5/5 column (Amersham Biosciences) equilibrated with buffer A. The protein was eluted with a linear gradient from 0 to 200 mmol/L NaCl, and stored at −80°C. Purity was confirmed by sodium dodecyl sulfate-polyacrylamide gel electrophoresis. In S100- A12 preparations used for this study, endotoxin levels were less than 0.1 pg/μg of S100A12 as measured by the limulus amebocyte assay (Bio Whittaker, Walkersville, Md, USA).

### Cell culture

Human monocytic THP-1 cells (American Type Culture Collection, Manassas, Va, USA) were cultured in RPMI 1640 supplemented with 10 vol% heat-inactivated fetal bovine serum (FBS) (Cansera, Etobicoke, ON, Canada), 100 U/mL penicillin and 100 μg/mL streptomycin (Invitrogen, Grand Island, NY, USA) at 37°C 5% CO_2_ in air.

### Cell migration assay

The migration of THP-1 monocytes was studied using a Chemotaxicell 24-well disposable chamber with 5 μm pores (Kurabo Industries Ltd, Osaka, Japan). Cultured cells were harvested and washed three times with Hank's balanced salt solution and then re-suspended in assay medium (RPMI 1640 with 0.5 vol% heat-inactivated FBS, 10 mmol/L N-(2-hydroxyethyl)piperazine-N'-(4-butanesulfonic acid), pH 7.4). Each drug and stimulant were premixed in the assay medium and incubated at 37°C for 30 min. Then 0.05 mL of the mixture was placed into the lower chamber contained 0.45 mL/well of the assay medium. Then 0.2 mL of cell suspension (6 × 10^5^ cells) was added to the upper chamber and incubated for 3 h at 37°C in 5% CO_2_. Cells passing through the membrane were collected from the lower well and counted with the EPICS XL/MCL flow cytometry system (Beckman Coulter, Fullerton, Calif, USA) followed by mixing of a predetermined number of flow-count fluorospheres (Beckman Coulter). The total number of cells that had migrated to the lower wells was determined by the ratio of cells to fluorospheres using the following formula: cells per microliter = [(cells counted)/(fluorospheres counted)] × fluorospheres/microliter. Each sample was assayed in triplicate.

### Statistical analysis

Data were presented as means ± SE. The Student *t* test following the F-test was used for analysis of differences between two groups. Multiple comparisons among treatment groups were assessed by one-way analysis of variance, followed by the Dunnett's test. Values of *P* < .05 were considered statistically significant. All statistical calculations were performed with the Statistical Analysis System (SAS Institute, Cary, NC, USA).

## RESULTS

### Effects of olopatadine on the migration of THP-1 monocytes induced by S100A12 or RANTES

**Figure 1:**
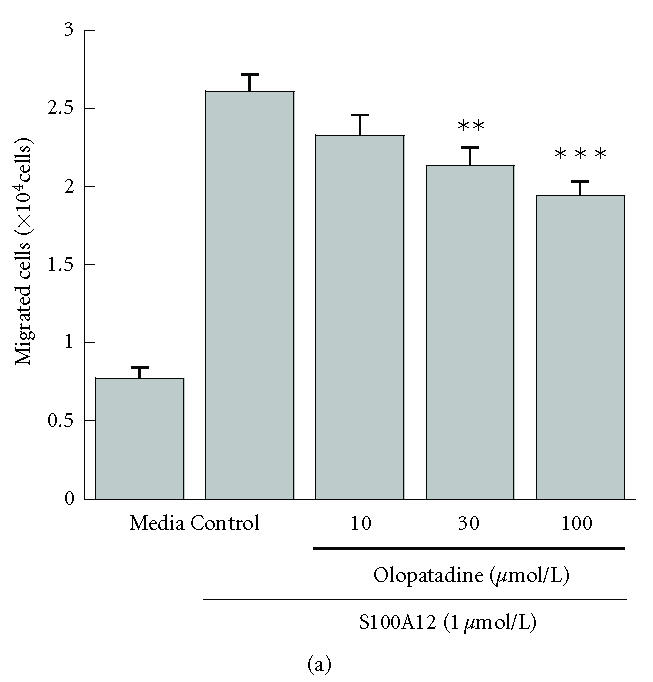

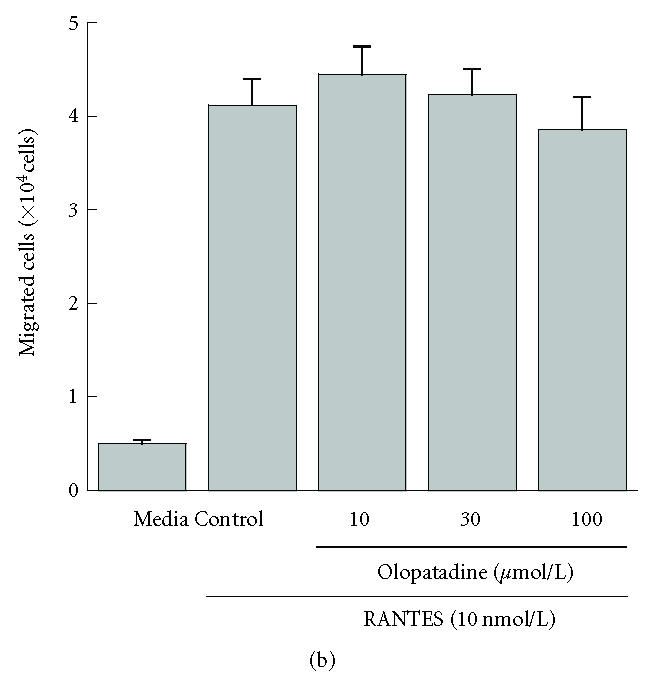
Effects of olopatadine on the migration of THP-1 cells induced by S100A12 (a) or RANTES (b). The numbers of cells migrating toward S100A12 (1 μmol/L) or RANTES (10 nmol/L) during 3 h incubation were determined by flow cytometry. The values in panel (a) are means ± SE from 3 independent experiments (*n* = 9), and those in panel (b) are means ± SE from 2 independent experiments (*n* = 6). **P < 0.01; ***P < 0.001 (versus control, Dunnett's test). Media, spontaneous migration.

The migration of THP-1 monocytes was studied in the presence of S100A12 or RANTES. S100A12 induced the migration of THP-1 cells at concentrations between 0.75 and 2 μmol/L (data not shown). Therefore, we examined the effect of olopatadine on the activity of S100A12 at 1 μmol/L, a submaximal concentration. After pretreatment of S100A12 for 30 min, 26095 ± 1044 cells/well migrated to the bottom chamber at 1 μmol/L of S100A12, indicating a 3.4-fold increase in comparison with the control value of 7699 ± 693 cells/well (Figure 1(a)). On the other hand, RANTES caused a 6.0-fold increase in the migration of THP-1 cells at 10 nmol/L (Figure 1(b)). As shown in Figure 1(a), pretreatment of S100A12 with olopatadine resulted in a concentration-dependent reduction of THP-1 cell migration induced by S100A12, although the reduction was only partial. The reduction of THP-1 cell migration by olopatadine is apparently not caused by inhibition of cell motility, since parallel studies showed that olopatadine had no effect on the migration of cells induced by RANTES (Figure 1(b)).

### Effect of amlexanox on THP-1 migration induced by S100A12

**Figure 2:**
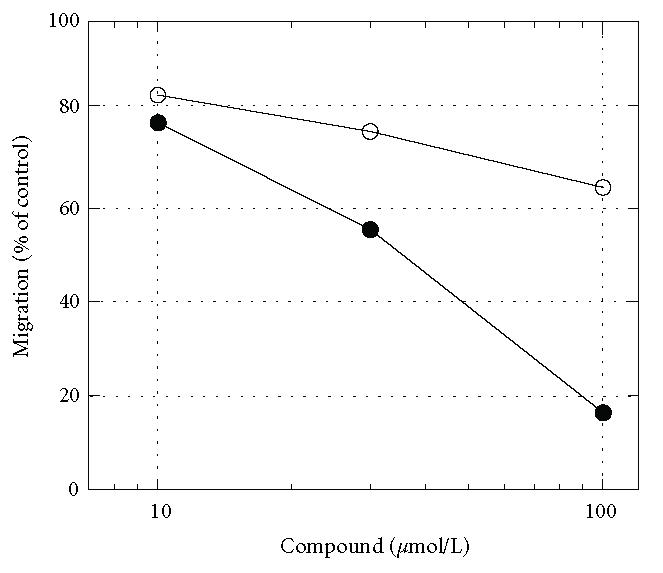
Comparison of the abilities of olopatadine (open circle) and amlexanox (closed circle) to inhibit THP-1 migration induced by S100A12. Migration assay was performed as described for the prior experiments. Values are percentages of control, and are the means of 2 independent experiments.

Amlexanox is also reported to bind to S100A12 [12]. In addition, it has already been reported that amlexanox binds to S100A13 and represses the FGF-1 release induced by S100A13 [13, 14]. Accordingly, it is expected that amlexanox also inhibits the function of S100A12. Figure 2 shows that amlexanox suppressed THP-1 migration induced by S100A12 more efficiently than olopatadine. While amlexanox suppressed the THP-1 migration induced by RANTES to some extent (data not shown), it was weak in this respect compared with inhibition of migration by S100A12. It is likely that amlexanox suppresses the effect of S100A12 by binding to S100A12 rather than by inhibiting cell motility.

### Effects of ketotifen on the migration of THP-1 cells induced by S100A12 or RANTES

**Figure 3:**
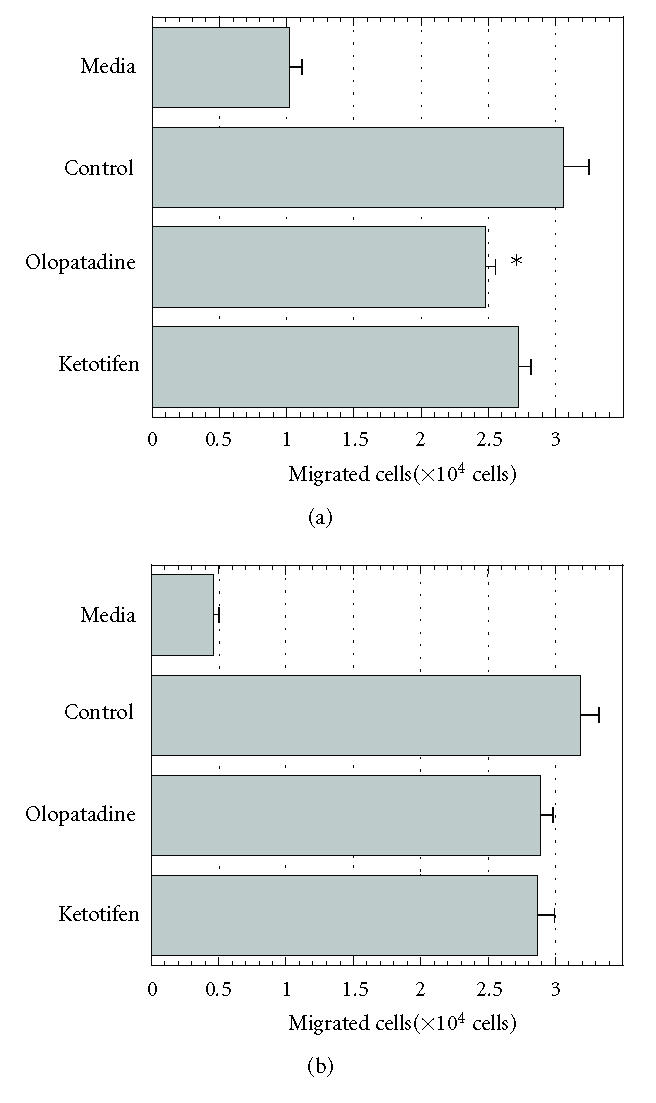
Effects of olopatadine or ketotifen on the migration of THP-1 cells induced by S100A12 (a) or RANTES (b). The numbers of cells migrating toward S100A12 (1 μmol/L) or RANTES (10 nmol/L) during 3 h incubation were counted. Values are means ± SE from 2 independent experiments (*n* = 6). *P < 0.05 (versus control, Student *t* test). Media, spontaneous migration.

Next, to determine whether histamine H_1_ receptor antagonistic activity of olopatadine is related inhibition of the activity of S100A12, ketotifen, another histamine H_1_ receptor antagonist, was tested. Figure 3 shows that ketotifen (50 μmol/L) did not affect THP-1 migration induced by either S100A12 and RANTES, though it has already been shown that ketotifen has more potent histamine H_1_ receptor antagonistic activity than olopatadine [21, 22]. These results suggest that olopatadine directly binds to S100A12 and inhibits its function rather than histamine H_1_ receptor antagonistic activity.

## DISCUSSION

S100A12 has been identified as a binding protein to antiallergic drugs, such as olopatadine, amlexanox, and cromolyn, using drug-affinity columns [19]. S100A12 has multiple inflammatory effects [8–11]. If these drugs suppress the activity of S100A12, a new anti-inflammatory mechanism of action of these drugs can be demonstrated. In the present study, we confirmed that S100A12 induced the migration of THP-1, a human monocytic cell line, at 1 μmol/L, and then that olopatadine and amlexanox suppressed this migration. Moreover, these drugs had little effect on the migration of THP-1 cells induced by RANTES, suggesting that olopatadine and amlexanox bind to S100A12 specifically and suppress its activity.

Previous studies showed that S100A12 induced chemotactic responses at much lower concentrations (0.01–1 nmol/L) [9, 10], whereas no response to S100A12 at lower concentrations was detected in our experiments. Recently, x-ray analysis has revealed two crystal forms of recombinant S100A12 [21]. Therefore, the effective concentration of S100A12 may vary according to experimental conditions. It is possible that in some conditions lesser amounts of drugs are sufficient for suppression of the activity of. In fact, it is necessary to examine the effect of olopatadine on the function of S100A12 in vivo experiments.

Ketotifen in contrast to olopatadine did not suppress the THP-1 migration induced by S100A12, suggesting that olopatadine may have the distinctive feature of direct inhibition of the function of S100A12. This new mechanism of action of olopatadine may in addition to its potent histamine H_1_ receptor antagonistic activity provide high degree of efficacy in the treatment of allergic diseases.
